# Measuring attentiveness toward oral care needs: a comparative study of Indonesian care workers in Japan and Indonesia

**DOI:** 10.1186/s12960-021-00614-y

**Published:** 2021-06-01

**Authors:** Yuko O. Hirano, Susiana Nugraha, Hiroyasu Shiozu, Misako Higashijima, Tri Budi W. Rahardjo

**Affiliations:** 1 Institute of Biomedical Sciences, Nagasaki University, 1-7-1 Sakamoto, Nagasaki, Nagasaki 852-8520 Japan; 2 Faculty of Health Sciences, University of Respati Indonesia, Jakarta, Indonesia; 3 Department of Occupational Therapy, Chubu University, Kasugai, Japan; 4 Faculty of Rehabilitation Sciences, Nishikyushu University, Kanzaki, Japan

**Keywords:** Older adults, Oral care, Care skills transfer, Indonesia, Japan, EPA care workers

## Abstract

**Background:**

Japan has opened its labor market to care workers from Indonesia under the Japan–Indonesia Economic Partnership Agreement (EPA). However, few studies have examined the types of care skills transferred between countries. We therefore analyzed Indonesian care workers employed in Japan and Indonesia to identify discrepancies in their attentiveness toward oral care in older adults.

**Methods:**

A checklist comprising 42 items of universal oral care assessment was developed prior to the study and distributed via the Internet to 418 Indonesian EPA care workers in Japan and via a paper survey to 213 Indonesian care workers in Indonesia. The Mann–Whitney *U* test was used to compare the distribution of scores for each checklist item for each group.

**Results:**

The respondents were 110 Japan-based EPA care workers (response rate: 26.3%) and 213 Indonesia-based care workers (response rate: 99.1%). Japan-based care workers were significantly more likely to perform environmental observations of their older adult patients (*p* < 0.001) and to check items on the oral care checklist during feeding (*p* < 0.001) and post-meal (*p* = 0.001), while Indonesia-based care workers were more likely to check the overall condition of patients before meals (*p* = 0.021).

**Conclusions:**

Discrepancies in checking oral care between the two groups were attributed to the differences in laws and regulations governing the care environments. Indonesian care workers employed in Japan have the advantage of learning to employ a more systematic approach in caring for older adults, in accordance with Japan’s Long-Term Care Act. This approach could contribute toward lowering the risk of aspiration pneumonia in Indonesia. A training program designed for returning migrant workers to transfer newly developed oral care skills will thus be essential for Indonesia to diminish the negative impacts of its aging population.

## Background

The movement of care workers across national borders is a global trend, with the Organization for Economic Co-operation and Development (OECD) reporting that more than 5.1 million labor migrants entered OECD countries on temporary migration programs in 2018, which represents a 5% increase over 2017 [1]. Japan is not an exception. Since 2008, Japan has recruited foreign care workers under economic partnership agreements (EPAs) with Asian countries, starting with Indonesia and followed by the Philippines (in 2009) and Vietnam (in 2014). The total number of care workers entering Japan from these three countries under EPAs was 5026, as of June 2019 [2]. One of the most important motivations of Indonesian care workers coming to Japan is to develop professionally and learn a high quality of care [3] based on Japan’s prolific experience in providing care for older adults. Indonesian care workers in Japan, especially those among the first EPA batch, experienced some friction because of the differences in the care cultures between the two countries; however, most of them were gradually able to adjust [4]. Japan offers unique learning opportunities for Indonesian care workers, who report high satisfaction with their employment in Japan [5]. Indonesian care workers tend to return to their home country after working in Japan for several years. Based on data publicized by the Ministry of Health, Labour and Welfare of Japan [6], we calculated that approximately 60.3% of care workers who take the test, pass the national board examination for certified care workers. This means that approximately 40% of Indonesian workers—those who failed and were therefore not qualified as a care worker—returned home, as per Annex 10 of the EPA [7]. Moreover, it is obvious from their narrative that most Indonesian returnees wish to contribute through the care skills that they obtained in Japan. In Hirano et al.’s [8] study, an Indonesian returnee reported that she surprised her family and friends by providing sufficient water for bedridden older adults, who showed remarkable revitalization afterward. This shows that Indonesian care workers are likely to learn Japan’s high quality of care for older adults and employ these skills when they return to Indonesia.

Although extensive research exists on the training of care workers worldwide, there are few studies involving migrant care workers [9]. Furthermore, most existing literature on migrant care workers [10] focuses on migrants’ continuous engagement in care work in destination countries, not their potential to transfer skills to their home country. Nonetheless, as per the World Health Organization’s Global Code of Practice on the International Recruitment of Health Personnel [11], it is essential for the destination countries to facilitate circular migration, so that the care skills migrant care workers obtain in the destination countries can be used to the benefit of both source and destination countries.

This study focused on the potential for the transfer of oral care skills, which are essential for satisfying the basic needs of care recipients, such as older adults. Oral care is the job most frequently conducted by care workers who entered Japan under EPAs [12]. The importance of oral care is underpinned by the association between pneumonia and oral health [13]. Moreover, when considering nursing for home-acquired pneumonia, which reflects a clinical environment similar to the one of the current study, professional oral care may reduce mortality due to aspiration pneumonia, compared with usual care [14].

Specifically, the transfer of oral care skills is important for older adults, who may be at risk of aspiration pneumonia, which is caused by a defective swallowing mechanism and occurs in patients with malfunctioning deglutition and cough reflex. Older adults are particularly vulnerable, with aspiration pneumonia being the fourth leading cause of death in Japan [15, 16]. Although there are no specific statistical data on aspiration pneumonia in Indonesia, it is likely present. As part of the effort to combat the negative effects of aging in society, Japanese researchers have developed various approaches to promote proper deglutition, which is influenced by age and bolus size [17] and is related to the level of nutrition and activities of daily living, particularly for older adults with sarcopenia [18]. Applying the appropriate trunk position was shown to be beneficial for shortening oral and pharyngeal transfer times in older adults with dysphagia in a clinical setting [19]. These findings suggest that aspiration pneumonia could be prevented via attentive intervention that focuses on maintaining a good posture and a bolus size appropriate for older adults.

Oral care in Japan has begun to evolve to strategically prevent aspiration pneumonia. We propose that improving the level of excellence in the care work for older adults must involve a combination of careful observation and medical knowledge. The effective management of dysphagia, for example, is particularly important for older adults recovering from a stroke or receiving treatment for dementia. Older adults who have experienced a right-hemisphere stroke are likely to ignore the left side of their vision field because of the malfunction in the part of the brain damaged by the stroke. Therefore, food that older adults with this condition cannot see is likely to remain uneaten; thus, care workers must alert their care receivers to the uneaten food or, occasionally, turn their plates around to help them to see all of their food.

The provision of training in techniques and skills that build upon evidence-based medicine are urgently required for care workers who aid older adults who are vulnerable to swallowing dysfunction. It is also crucial that care environments for older adults provide appropriate oral care practices, such as providing food that care receivers can easily cut to a proper bolus size for swallowing, helping them maintain a good posture when eating, and rearranging food items to maximize their visual attention. We suggest that the proper adoption of these skills may contribute to lower the risk of aspiration pneumonia in Indonesia, where the population is expected to age rapidly in the coming decades.

The current global era of care worker migration offers high potential for the efficient transfer of care skills between care workers and among countries. However, few studies have examined the types of care skills that foreign care workers could learn abroad and apply on returning to their country of origin. Therefore, we investigated the potential for transferring oral care skills from Japan to Indonesia for the prevention of aspiration pneumonia.

## Methods

We studied two groups of care workers for older adults who shared the same nationality (Indonesian) but worked in different countries (Indonesia and Japan) to decrease the influence of cultural or societal context on our findings. To understand the oral care settings in Japan and Indonesia, both participant groups were asked to verify the essential points of which they were aware during the provision of oral care for older adults.

### Instrument

The list to check the essential points for oral care (hereafter, checklist) was developed by the following procedure.

First, to satisfy the construct validity of the checklist and confirm that the checklist contained the entire process of ingestion, 62 items were developed in Japanese by one of the authors (HM), based on Leopold’s Five-Stage Process of Ingestion [20], which consists of the anticipatory, preparatory, lingual, pharyngeal, and esophageal stages. Each stage is compatible with the five periods under the second domain developed by the authors, as shown in Table 1.

**Table 1:** Comparison of oral care checklist score between the two study groups

No.	Observation item	Indonesian care workers in Japan (*n* = 110)	Indonesian care workers in Indonesia (*n* = 213)	*P value*
I	Environmental observation			
1	There is a separation between the dining room and the bedroom	96%	55%	< 0.001
2	Adjustable dining tables or special chairs that can be used according to need are available for personal use	91%	47%	< 0.001
3	There are applicable utensils (e.g., spoons and chopsticks) that can be used in accordance with the impairment of the individual user*	98%	–	
4	There are variations in the form of food (e.g., porridge cut into bite-size pieces and soft chopped food)	98%	64%	< 0.001
5	There is portable mucus suction	77%	54%	< 0.001
6	There is a tool that can be used by care workers or patients to notify other care workers if there is an emergency (e.g., a bell).	89%	44%	< 0.001
7	Numbers of care workers and older adults are balanced when conducting meal supervision	66%	52%	0.008
8	There is an allocation of one care worker for every care receiver who requires total care**	28%	60%	< 0.001
9	The care workers understand the dietary needs of each receiver	78%	74%	0.257
	Total score	6.2 (SD:1.4)	4.5 (SD:1.9)	< 0.001
II	Observation of physiologic functions and eating capabilities			
A	Overall condition before meal			
1	Older adult moves differently than usual	72%	58%	0.009
2	Older adult is in poor condition/sleep deprivation	42%	57%	0.006
3	Older adult has fever	20%	50%	< 0.001
4	Older adult is in a state of coughing.	27%	43%	0.004
5	Older adult has different blood pressure (higher or lower) and pulse (bradycardia, tachycardia) than usual	36%	49%	0.012
	Total score	2.0 (SD:1.4)	2.6 (SD:2.0)	0.021
B	Meal preparation period: Waiting time before arrival of food and serving of food			
1	Older adult cannot sit in a stable position	66%	54%	0.018
2	Older adult is not fully conscious	39%	41%	0.428
3	Older adult appears calm	40%	55%	0.008
	Total score	1.5 (SD:1.2)	1.5 (SD:1.2)	0.839
C	Feeding period:			
1	The dining table cannot be set up in accordance with the bodily positions of older adult	82%	49%	< 0.001
2	There are difficulties when eating (e.g., cannot use a spoon for food or hold food)	79%	65%	0.007
3	There are problems in paying attention to food and the environment	77%	55%	< 0.001
4	There is a problem when placing food into the mouth (e.g., hand vibrates or food falls)	72%	67%	0.207
5	There is a speed problem when placing food into the mouth.	72%	55%	0.003
6	There is a problem with the amount of food that is placed into the mouth	68%	55%	0.014
7	Older adult cannot ask for help when having difficulties during the eating process	55%	44%	0.035
8	Older adult refuses to be helped when eating (e.g., does not want to open the mouth or feed themselves)	63%	49%	0.014
	Total score	5.7 (SD:1.9)	4.4 (SD:2.9)	< 0.001
D	Swallowing period:			
1	Older adult cannot take in food smoothly (e.g., lips cannot close or food falls out of the mouth)	58%	54%	0.302
2	There are problems with chewing (e.g., lack of chewing, removing or leaving hard food, very fond of eating soft food only, or chewing for a long time)	70%	66%	0.287
3	Older adult cannot chew food into appropriate shapes and sizes to be swallowed	59%	59%	0.52
4	Older adult hoards food in the mouth (e.g., stores food in the mouth but does not swallow)	65%	57%	0.083
5	There are problems in the process of swallowing food (e.g., cannot swallow food or takes time to swallow food)	71%	54%	0.002
6	There is a swallowing disorder (e.g., food cannot be channeled into the esophagus)	48%	48%	0.535
7	There is a sound of fluid in the esophagus	43%	47%	0.3
8	Older adult chokes when eating	59%	56%	0.362
9	Older adults does not exhibit coughing when choking	39%	54%	0.009
10	Older adult takes a lot of time from start to finish when eating	81%	60%	< 0.001
11	Older adult looks tired when eating	48%	49%	0.503
12	Older adult looks weak when eating, and cannot maintain proper posture (e.g., the body position is always slumped)	66%	53%	0.018
13	Older adult does not finish one portion of provided food	72%	55%	0.002
	Total score	7.8 (SD:3.7)	7.1 (SD:4.5)	0.216
E	Post-meal period			
1	Older adult does not brush teeth after meals	41%	32%	0.07
2	There is shortness of breath after meals	29%	45%	0.005
3	Older adult cannot use toothbrush	66%	40%	< 0.001
4	There is leftover food after brushing teeth	69%	40%	< 0.001
	Total score	2.1 (SD:1.3)	1.6 (SD:1.2)	0.001

Scoring system: 1 = yes; 0 = no

^*^This item was omitted from the questionnaire for Indonesian care workers in Indonesia

^**^This item was translated “There are special nurses who are responsible for older adult with total care” in the Indonesian version to suit the environment of the care setting in Indonesia

Second, the 62 items were translated by one of the authors (NS), who can speak both Japanese and Indonesian, and an Indonesian bilingual translator. The two worked independently to translate the Japanese checklist. One of them translated the original Japanese version to Indonesian and the second translated the Indonesian version back into Japanese; the process was blinded and the second translator did not see the original Japanese version beforehand. The two Japanese versions (original and back-translated) were then checked for discrepancies. Any inconsistencies were discussed with another Indonesian bilingual translator engaged in care work in Japan, and consensus was reached.

Third, a multidisciplinary meeting was held to select items from the checklist that satisfied the analyzed care environments and observe the conditions of care receivers to maintain safe ingestion. The original checklist was intended for use prior to care provision in Japan; therefore, we chose the items indifferent to the cultural differences between Japan and Indonesia. The multidisciplinary team consisted of three nurses (NS and two others, one of whom was a Japanese nurse speaking both Indonesian and Japanese), an occupational therapist (HM), a medical sociologist (HY), an anthropologist, two nutritionists, seven gerontologists, three dentists (TR and another two), and three pedagogical experts from both Indonesia and Japan. We also invited two Indonesian care workers who had previously worked in Japan to our meeting as observers. Based on the discussions between the participants, of the 62 items from the original checklist, 42 were selected after adjusting them for the socio-cultural conditions in Indonesia and divided into two domains (Table 1): (1) observation of the care setting environment and (2) observation of functions and eating capabilities. The first domain contained nine observational items regarding the safety of the care environment for older adults’ dietary needs. The second domain was divided into five periods: (A) overall condition of the patient before meal (five items), (B) meal preparation period (three items), (C) feeding period (eight items), (D) swallowing period (13 items), and (E) post-meal period (four items).

Fourth, language appropriateness was discussed to confirm content validity. Two nurses, two dentists, and a care worker engaged in care work in Japan (including two of the authors: NS and TR) tested the content validity.

Finally, a confirmatory factor analysis was conducted to test stability. The results derived six factors, which were almost the same as the originally developed six categories, comprising the first domain and five periods under the second domain. Internal consistency reliability was tested using Cronbach’s alpha for each category; values ranged from 0.541 to 0.896.

A self-report questionnaire that included the oral care checklist and demographic characteristics of the respondents (i.e., gender, age, years’ experience in care work, and occupation at the care facility) was used and the respondents were asked to provide yes/no responses (yes = 1, no = 0) to indicate whether they were attentive to the necessity of care intervention for each item.

### Participants

Eligible participants were Indonesian care workers who worked in public and private care facilities in Indonesia and Japan. In Indonesia, nonprobability purposive sampling was performed to select the participants. In total, 215 care workers from 18 care facilities (7 government-owned and 11 privately owned) from four prefectures (Yoghakarta, West Java, DKI Jakarta, and Tangerang Banten) were enrolled in the study and 213 completed the survey. All participants, including 52 nurses, were engaged in care work. We compared the participants by the country in which they engaged in care work, after confirming there were no laws and regulations that restricted the job description of the nurses engaged in care work in Indonesia. We conducted a separate statistical analysis to test the distribution of attentiveness in care by occupational title. There were no statistical differences in attentiveness in care between nurses and non-nurses based on domain/period-based total scores. Therefore, in the latter part of this study, all observations from Indonesia (Indonesia-based care workers) were tested, regardless of the occupational title. In Japan, 418 Indonesian care workers who entered Japan under the certified care worker track designated by the Japan–Indonesia EPA (Japan-based EPA care workers) were recruited through social networking services (Facebook). In total, 110 completed the survey. Due to EPA regulations, all 110 participants enrolled in the study were nursing graduates engaged in care work, as follows: 57 (51.8%) in special nursing homes, 33 (30%) in healthcare facilities for older adults, and 20 (18.1%) in other types of care institutions, including hospitals and special care facilities for people with disabilities.

### Data analyses

Scores for each oral care item were added by domain/period and a within-group analysis of differences between the added scores was conducted using the demographic characteristics of the respondents. Mann–Whitney *U* tests and Chi-square tests were conducted to compare the distribution of each item between Indonesia-based care workers and Japan-based EPA care workers at a *p* < 0.05 significance level. We used IBM SPSS Statistics for Windows version 25J for the statistical analyses.

## Results

### Descriptive statistics

Among the total 213 (response rate: 99.1%) Indonesia-based care workers and 110 (response rate: 26.3%) Japan-based EPA care workers, the average age was significantly higher among the former (35.2 years; standard deviation [SD] = 11.2), compared with the latter (27.9 years; SD = 3.1; *p* < 0.001), as was the duration of work experience (7.3 years, SD = 6.6 vs. 3.7 years, SD = 2.8, respectively; *p* < 0.001). There was no significant difference in gender distribution by group.

### Cross-sectional analysis of respondent characteristics and oral care domain scores

The domain/period-based total scores for Indonesia-based care workers were tested by institution (public or private) and type of work (nursing or other care work), and no significant correlations were found. For Japan-based EPA care workers, we tested the domain/period-based total scores by the type of institution (working in a special nursing home or not), clinical experience as a nurse prior to entering Japan, completion of a care worker training course while in Indonesia, and experience as a care worker prior to entering Japan, and found no significant correlations.

### Comparisons between Indonesia-based and Japan-based EPA care workers

Comparisons of each item on the oral care checklist between Indonesia-based and Japan-based EPA care workers are shown in Table 1. The results indicate that the latter were more likely to perform environmental observations (*p* < 0.001), especially during both feeding (*p* < 0.001) and post-meal (*p* = 0.001). Conversely, Indonesia-based care workers were more likely to check the overall condition of the patient before meals (*p* = 0.021). This was particularly true for the following items: “older adult is in a poor condition/sleep deprivation” (*p* = 0.006), “older adult has a fever” (*p* < 0.001), “older adult is coughing” (*p* = 0.004), and “older adult has different blood pressure (higher or lower) and pulse (bradycardia, tachycardia) than usual” (*p* = 0.012). To determine whether a nursing background contributed to these differences, we compared the proportions of Indonesia- and Japan-based EPA care workers with nursing backgrounds, and found no significant difference between groups.

## Discussion

Providing care skills training helps not only care recipients, but also care workers [8]. When training migrant care workers, it is essential to consider cultural diversity of care [21]. This study statistically demonstrated that, even among care workers of common nationality (Indonesian), how care workers provided care varied, because the manner in which care is applied is subject to the environment and care setting. Therefore, analyzing discrepancies in attentiveness between Japan-based Indonesian EPA care workers and Indonesia-based care workers may bridge the gaps in the provision of care for older adults between Japan and Indonesia, which could result in improving good practice guidelines for the transfer of care skills.

We compared the quality of oral care provided to older adults by Indonesia-based care workers and Japan-based EPA care workers. Although all participants were Indonesian, there were differences between the oral care item groups checked by them. Apart from the meal preparation and swallowing periods, there were other significant differences between groups. Specifically, Japan-based EPA care workers were more likely to make environmental observations during feeding and post-meal, whereas those based in Indonesia were more likely to make observations about the overall condition of their patients before meals. We offer three possible interpretations for these discrepancies.

First, the working environments in these two countries are different. Generally, care workers in Japan have higher caseloads than their counterparts in Indonesia and are also expected to simultaneously provide care for several older adults. This might have prevented the Japan-based EPA care workers from focusing on the overall condition of each patient prior to meals. Conversely, the ratio of care workers to older adults is higher in Indonesia compared with Japan, thereby allowing for more time to carefully observe each patient.

Second, regulations that dictate the minimum physical requirements for the institution-based care of older adults differ between the two countries. Japan-based EPA care workers are expected to follow all rules and regulations established in Japan, with care facilities required to uphold ministerial ordinances. Specifically, Ministerial Ordinance No. 39 (enacted on March 31, 1999) states that food should be provided in a form that meets the physical requirements of each individual older adult, which is crucial for dysphagia management (i.e., postural adjustments, swallowing maneuvers, and diet modifications) [22–24]. This ordinance has directly led to improvements in the care environment and skill sets of care workers in Japan; by contrast, there is no comparable regulation in Indonesia.

Third, the formal job description and employer expectations of care workers differ between Japan and Indonesia. Having no specific legislation to regulate the role of a care worker in Indonesia, all care workers share common tasks, regardless of their occupational title. Therefore, Indonesia-based care workers were comparatively more attentive to the overall condition of their older adult patients, particularly during the pre-meal period, including checking vital signs such as blood pressure and assessing sleep deprivation, fever, and cough. In Japan, such tasks are often under the purview of nurses, where the job descriptions of nurses and care workers are clearly delineated by law. Under this system, a nurse is defined as someone who is licensed under the Ministry of Health, Labour and Welfare of Japan to provide medical treatment or assist in providing medical care for people who are injured, ill, or recovering from childbirth [25]. The role of a care worker is broader: a “care worker must in good faith engage in the services so as to allow the persons under their charge to maintain personal dignity and live an independent life in light of their standing at all times” [26]. In accordance with these laws, the duties of care workers in Japan are clearly divided: nurses check vital signs (because this is a form of medical care) and care workers assist older adults with activities of daily living that are important to maintain quality of life.

The strengths of the institution-based care environment in Japan were reflected in the differences in the practical application of oral care skills between our two study groups. The environmental observations made during the feeding and post-meal periods enabled the Japan-based EPA care workers to be more attentive in helping older adults maintain not only oral functioning, but also good posture and motion while eating. Care skills to assist older adults with maintaining a good posture while eating can help prevent dysphagia and decrease pharyngeal residue, including the “chin down technique” and “head rotation technique”, which minimize dysphagia risk by changing the angle of the head [27–30]. Further, the authors have routinely observed Japanese care workers instructing their Indonesian EPA counterparts on performing these skills.

Another beneficial practice that Indonesian care workers appeared to learn from working in Japan was greater attentiveness to oral care needs, particularly during the post-meal period. Post-meal oral care, such as tooth brushing, is also useful for dysphagia prevention, as reported by Yoneyama et al. [31, 32]. More than 65% of the Japan-based EPA care workers in this study engaged in oral care post-meal, regardless of care institution. We propose that Indonesian EPA care workers could help their compatriots by transferring these skills to the care setting in Indonesia.

Currently, older adults represent 9.6% of the Indonesian population (268 million) and the older-age dependency ratio (age 60 + /age 15−59) is 15.01% [33]. The workload of care is sufficiently low to allow them to be flexible when providing care. However, it has been predicted that, by 2045, the older segment of the Indonesian population will increase to 19.8%, with the older-age dependency ratio increasing markedly to 24.5% [34]. Therefore, in the near future, Indonesian society may face some of the issues that Japan is currently experiencing. Our results suggest that Indonesia may benefit from learning some of Japan’s recent lessons.

There are important factors to consider when attempting to transfer care skills and practices between these two countries. First, we must remain aware that care work varies in accordance with the cultural and societal setting. As indicated above, there are significant discrepancies between Japan and Indonesia in terms of provision and regulation of care, job descriptions for nurses and care workers, and responsibility distribution between care providers. Our study identified areas that require focus to bridge the gaps in older-age care provision between Japan and Indonesia, assuming such discrepancies reflect cultural and societal differences in care work. Accordingly, it is necessary to adopt a multidisciplinary approach to develop a training module for oral care that involves medical professionals, sociologists, and anthropologists. Second, a discussion on the governance of returning migrant workers is a prerequisite for the dissemination of such training module. Indeed, our ultimate goal is to contribute not only to the well-being of older adults in Indonesia, but also to that of its returning migrant workers. As recommended by the Global Forum on Migration and Development [35], bilateral circular labor agreements should include the country of origin’s responsibility for the recognition of workers’ skills when they return. Accordingly, as part of the Law on Protection of Migrant Workers (No. 18/2019) [36], the National Agency for the Protection and Placement of International Migrant Workers of Indonesia initiated a program under which returning migrants, particularly those who have engaged in care work in Japan, will train local care workers and Japan-bound Indonesian care workers. Therefore, establishing a system to maximize the experiences of care workers as they return from Japan could facilitate the effective transfer of oral care skills to Indonesia in the near future.

### Limitations

There are some limitations to this study. First, the response rate for the Japan-based EPA care workers was low, which made sampling bias unavoidable. Second, the sample was limited to institution-based care workers. Further research is required to evaluate the oral care skills of home-based care workers.

## Conclusions

A novel oral care checklist was developed that revealed differences in oral care practices for older adults between care workers based in Indonesia and Indonesian EPA care workers based in Japan. Based on these findings, our final goal is to develop a new training program to help bridge the gaps in oral care provision between these two countries. Implementing such a training program in Indonesia would reduce the risk of dysphagia among older adults, which may be a cause of aspiration pneumonia. This is a small but significant step to transfer care skills that benefit both the destination and source countries of migrant care workers.

## Data Availability

The datasets generated and/or analyzed during the current study are not publicly available because of ongoing data collection. However, data will be released upon completion of the study and are available from the corresponding author on reasonable request.

## Abbreviations

EPA: Economic Partnership Agreement
SD: Standard deviation
